# Scalp electroacupuncture targeting the VTA^DA^ neurons to relieve negative emotions and promote the alleviation of chronic pain

**DOI:** 10.3389/fnins.2023.1323727

**Published:** 2023-12-20

**Authors:** Yanan Yang, Xiali Wei, Jun Tian, Ye Zhu, Shaohui Jia, Qing Shu

**Affiliations:** ^1^Department of Traditional Chinese Medicine, China Resources & Wugang General Hospital, Wuhan, China; ^2^College of Sports Medicine, Wuhan Sports University, Wuhan, China; ^3^Department of Rehabilitation, Zhongnan Hospital of Wuhan University, Wuhan, China

**Keywords:** chronic pain, negative emotions, electroacupuncture, reward, acupoint selection

## Abstract

**Object:**

Chronic pain and negative emotions are often linked, and both can impact the reward circuit. The use of electroacupuncture (EA) has been found to regulate and improve these conditions. This study explores the potential mechanism of chronic pain relief by adding acupoints with emotional regulation effect to the basis of routine EA analgesia, to optimize the acupoint compatibility scheme of EA in the treatment of analgesia.

**Method:**

For this study, 42 male Wistar rats were used. Recombinant adeno-associated viruses were used to label and regulate the activity of dopamine (DA) neurons. The rat model was established by complete Freund’s adjuvant (CFA). Lower limb electroacupuncture (LEA) was applied to the ST36 and BL60 acupoints. In addition, LEA + scalp EA (SEA) was given using the GV20 and GV24+ acupoints besides ST36 and BL60. To evaluate the pain threshold, we measured 50% paw withdrawal thresholds and thermal paw withdrawal latencies. Negative emotions were evaluated through the open field test, marble-burying test, sucrose preference test, and forced swimming test. Moreover, the conditional place preference test was conducted to measure the reward behavior in response to pain relief. Immunofluorescence staining, Western blotting, and qPCR were used to detect the activity of the VTA^DA^-NAc reward circuit.

**Result:**

The injection of CFA significantly lowered the pain threshold. As the pain persisted, the anxiety and depression-like behaviors escalated while the response to reward reduced. Meanwhile, the VTA^DA^-NAc pathway was suppressed with pain chronification. However, activating DA neurons in VTA attenuated the effects induced by CFA. LEA could relieve chronic pain, negative emotions, and reward disorders, while also activating the VTA^DA^-NAc pathway. In addition, LEA + SEA exhibited a more pronounced effect compared with LEA alone. Nevertheless, chemogenetic inhibition of DA neurons decreased the efficacy of LEA + SEA in the treatment of chronic pain and associated comorbidities.

**Conclusion:**

Adding SEA to conventional LEA effectively alleviates negative emotions and chronic pain, potentially due to the activation of the VTA^DA^-NAc reward neural circuit. Thus, LEA + SEA is a more effective treatment for hyperalgesia and associated negative emotions compared with LEA alone.

## 1 Introduction

Chronic pain is pain that lasts or recurs for no less than 3 months and hence lacks early-warning physiological protective function (Woolf, 2010; Treede et al., 2015). It was reported that in the United States, 50.2 million adults accounting for nearly 20.5% of all suffered from chronic pain which is undoubtedly linked to reduced quality of life and a greater need for medical care (Yong et al., 2022). Nevertheless, clinical treatment is challenging because of the pain–emotion dyad. Studies demonstrated that chronic pain is characterized by negative emotions because they involve similar brain regions and neuronal networks (DosSantos et al., 2017). In addition, the newly revised definition of chronic pain by the International Association for the Study of Pain (IASP) also connects it to emotional experience (Raja et al., 2020). Based on different estimations, the prevalence of emotional disorders exceeds 85% in chronic pain sufferers (Hooten, 2016), whereas the presence of chronic pain ranges from 1 to 50% in individuals suffering from emotional disorders (Bair et al., 2003; Williams et al., 2003). Chronic pain and negative emotions usually reinforce each other, since negative emotions can also lead to increased pain perception and hinder pain relief (Meints and Edwards, 2018; Timmers et al., 2019). In clinical application, anxiolytics and anti-depression drugs are recommended for analgesia, indicating the importance of emotional regulation in the treatment of allodynia (Qaseem et al., 2017; Park et al., 2018). Considering the side effects brought about by medication, nonpharmacologic therapy has great potential in analgesia (Qaseem et al., 2017).

The mesolimbic dopamine (DA) system regulates motivation and incentive, which are essential to survival, by controlling individuals’ reactions to reward stimuli. Pain is similar to other aversive states, and the relief of pain is rewarding (Navratilova and Porreca, 2014). Consistent with the use of opioids, the DA reward system can be activated by pain stimuli, and adaptive changes in neuroplasticity of the reward circuit occur under the chronification of pain (Serafini et al., 2020). It has been pointed out that the DA system modulates emotions and pain perception, and the brain regions responsible for the aversive aspect of pain partly overlap with the reward circuit (Zhuo, 2016; Li et al., 2017; Liang et al., 2020), highlighting the critical role of the DA reward circuit in the pain–emotion dyad. Originating from the ventral tegmental area (VTA), the DA neurons mainly project to the nucleus accumbens (NAc), where the core is associated with motor function and the shell with reward (Groenewegen et al., 1996; Reed et al., 2015; Watanabe and Narita, 2018). In addition, the NAc shell (AcbSh) can be subdivided into dorsomedial, ventromedial, and ventrolateral regions according to its connectivity with subnuclei of other brain regions (Gibson et al., 2019), whereas the medial AcbSh was reported to activate reward seeking with the increase in DA levels (Reed et al., 2015; Richard and Fields, 2016).

As a traditional Chinese medicine modality, electroacupuncture (EA) has attracted the attention of scientists for the treatment of chronic pain owing to its safety and effectiveness (Li et al., 2019, 2020). Emerging evidence has proven the efficacy of EA in analgesia in both experimental and clinical research (Patel et al., 2020; Mao et al., 2021). Meanwhile, multiple studies showed that EA contributed to the relief of emotional disorders, with a high acupoint selection frequency on the head (Lin et al., 2020; Shen et al., 2020; Yin et al., 2022; Wei et al., 2023). In a study on drug addiction, the reward circuit was reported to be regulated by EA (Lee et al., 2021), but its effects on the reward circuit in the field of analgesia remain to be explored. Therefore, this study aims to investigate whether adding scalp EA (SEA) to conventional analgesia EA which focuses on acupoints in the lower limb can enhance the efficacy of lower limb EA (LEA) in treating emotional disorders and hyperalgesia. The proposed mechanism involves the activation of the VTA^DA^-NAc reward circuit.

## 2 Materials and methods

### 2.1 Animals

Forty-two male Wistar rats (250–300 g) were obtained from Si Pei Fu Biotechnology Co., Ltd. Animal experiments were approved by the Experimental Animal Welfare Ethics Committee, Zhongnan Hospital of Wuhan University (Approval No. ZN2023057) ***(Sup. 1)***. Rats were housed in cages with soft padding in the Experimental Animal Center, Zhongnan Hospital of Wuhan University. Before manipulation, the rats were adaptively fed for at least 7 days in a stable environment (22 ± 2°C, 50 ± 10% humidity) on a 12/12-h light/dark cycle with food and water *ad libitum*, continuous ventilation, and air filtration. The experiment strictly followed the guidelines of the National Institutes of Health for the care and use of laboratory animals.

### 2.2 Stereotaxic injection

**Figure 1:**
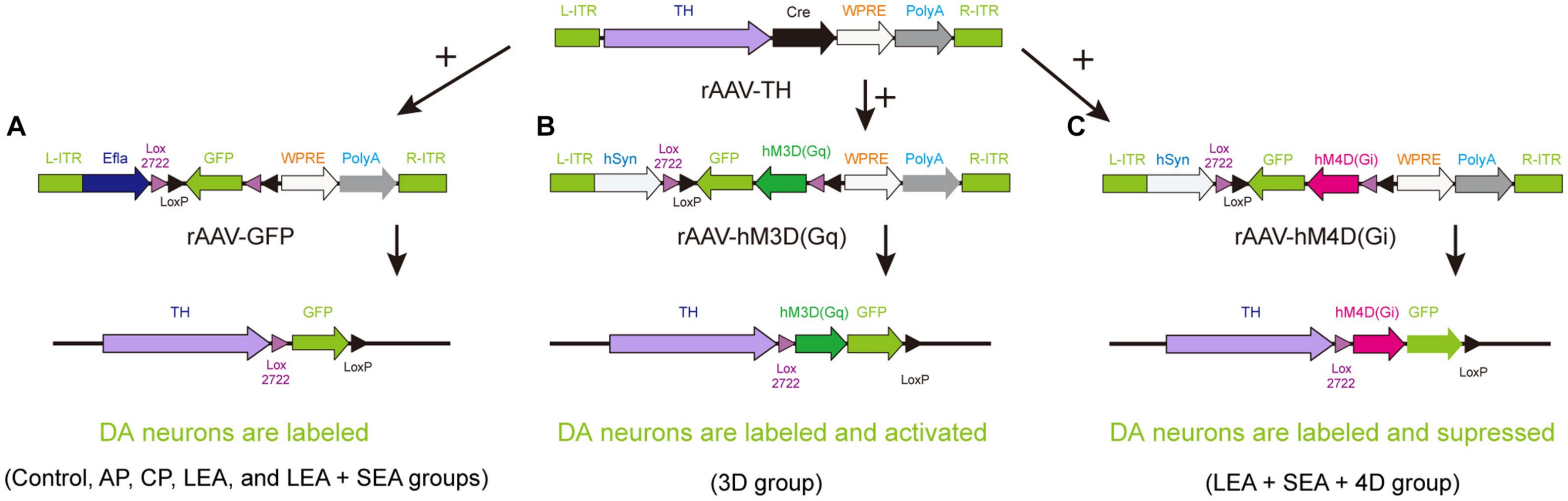
A strategy for combining viruses. **(A)** DA neurons are simply labeled by GFP in the control, AP, CP, LEA, and LEA + SEA groups. **(B)** DA neurons of rats in the 3D group are activated chemogenetically and labeled by GFP. **(C)** DA neurons are suppressed chemogenetically and labeled by GFP in the LEA + SEA +4D group. DA, dopamine.

Rats were anesthetized with isoflurane (3.5–4% for induction, 1.5–2% for maintenance) and fixed on a rat stereotaxic apparatus (RWD, Shenzhen, China). The hair on the head was shaved and the eyes were covered with erythromycin eye ointment to avoid corneal injury elicited by strong light. According to bregma and lambda points, the precise location of the VTA was determined for injection (AP, −5.5 mm; ML, ±1 mm; DV, 8 mm) (Taylor et al., 2015). An electric drill was used to make a hole in the skull, through which a total of 260 nL recombinant adeno-associated virus (rAAV) was vertically injected into the VTA at a rate of 26 nL per minute by glass microelectrodes (BF-100-58-10, Sutter Instrument Company) with an injection pump control. For monosynaptic tracing of DA neurons, rAAV-TH-CRE-flag-WPRE-pA (rAAV-TH) combined with rAAV-Efla-DIO-EGFP-WPRE-pA (rAAV-GFP) was applied to the control, acute pain (AP), chronic pain (CP), LEA, and LEA + SEA groups (Figure 1A). In the 3D group, rAAV-TH and rAAV-hSyn-DIO-hqM3D (Gq)-EGFP-WPRE-pA (rAAV-3D-GFP) were injected to trace and activate DA neurons (Figure 1B), while rAAV-TH combined with rAAV-hSyn-DIO-hM4D (Gi)-EGFP-WPRE-pA (rAAV-4D-GFP) was used to trace and inactivate DA neurons in the LEA + SEA +4D group (Figure 1C). During the surgery, the body temperature was kept around 37°C by a heating pad.

### 2.3 Establishment of rat model

Rats were anesthetized under light isoflurane, and the left hind paws were subcutaneously disinfected with 1% Iodophor. Between the second and third metatarsals, 100 μL complete Freund’s adjuvant (CFA, Sigma-Aldrich, F5881) was injected to establish the acute pain model (AP model, early time points post-CFA) and chronic pain model (CP model, late time points post-CFA). An equal volume of 0.9% saline was injected into the control. The injection point was pressed for 3 min to avoid CFA overflow.

### 2.4 Pain thresholds

#### 2.4.1 Pain withdrawal thresholds (PWTs)

A series of Von Frey hairs (37,450, Ugo Basile) was used to determine the 50% PWTs. During the test, rats were placed on barbed wire to adapt to the new environment for 20 min until they stopped exploring and grooming. Based on the up-down method introduced by Dixon and Chaplan (Dixon, 1980; Chaplan et al., 1994), filaments classified as 0.4 g, 0.6 g, 1 g, 2 g, 4 g, 6 g, 8 g, 15 g, and 26 g were chosen in this test, and it was started at 4 g. The 50% PWT was calculated as follows: 50% PWTs (g) = 10^[Xf + Kδ]^ / 10,000, where Xf is the value of the final filament used, K is the tabular value for the pattern of positive or negative reaction, and δ = 0.224 here.

#### 2.4.2 Thermal paw withdrawal latencies (PWLs)

To determine the PWLs, the rats were introduced to an intelligent hot plate instrument (RB 200, Techman, Chengdu) with hind paws put on the surface of the plate, the temperature of which was set at 52°C. The pain threshold and PWLs were determined based on the left hind paw’s duration of stay on the plate. PWLs were recorded on the same day as PWTs.

### 2.5 Behavior assessment

All assessments were conducted in a dimly lit environment (20–24°C, 40–60% humidity). Before the assessment, rats were placed in the test environment for at least one day to adapt. Assessments were started after intraperitoneal injection of clozapine-N-oxide (CNO) or 0.9% saline for 30 min.

#### 2.5.1 Open-field test (OFT)

The OFT was conducted in a 1 × 1 m open field with a camera on top to record behavior. The rats were placed in the center of the field and allowed to explore for 30 s. Behavior was recorded for the next 5 min. The field was divided into 16 equally sized squares by SuperMaze software (V 3.3.0.0, Shanghai Xinruan Information Technology Co., Ltd.). The central four squares were defined as central zones, while the outer 12 squares were defined as thigmotaxic zones. Frequency, distance of movement, and time spent in central zones and thigmotaxic zones were recorded and their ratio was used to assess anxiety-like behavior. Before the assessment, the field was cleaned with 10% alcohol to eliminate the odor of the previous rat.

#### 2.5.2 Marble-burying test (MBT)

For the MBT, nine marbles with a diameter of 2.5 cm were arranged in a 3 × 3 layout in a cage that was covered by cottonwood padding with a thickness of 5 cm. The number of marbles buried in 15 min was recorded, with more burials representing a higher tendency of anxiety. Marbles were only considered as buried if at least two-thirds of the volume was buried by padding.

#### 2.5.3 Sucrose preference test (SPT)

The SPT took five days. First, rats were trained to habituate to the 1% sucrose solution. On the first day, two bottles of 1% sucrose were provided to rats. After 24 h, one bottle of sucrose solution was replaced by pure water. Before measurement, rats were deprived of water for 24 h, and on the following day, all rats were given access to a bottle of sucrose solution and a bottle of pure water that were pre-weighed. Notably, the position of the two bottles was switched to avoid place preference bias. The consumption of water and sucrose was calculated on the next day. Sucrose preference was calculated as follows: sucrose preference (%) = consumption of sucrose solution / (consumption of sucrose solution and pure water) × 100%. A lower sucrose preference indicates a reduced ability to experience pleasure.

#### 2.5.4 Forced swimming test (FST)

The FST is widely used to examine susceptibility to depression (Yankelevitch-Yahav et al., 2015), which is assessed by determining the lack of ability to handle stress. The test was implemented with a SuperFst system (XR-XQX201, Shanghai Xinruan Information Technology Co., Ltd.), consisting of a video tracking system, Visu Track V 3.0 analysis software, and a vertical transparent cylinder containing water at a temperature of 24 ± 1°C. Rats were forced to swim in the inescapable container for 6 min, and only behaviors of the last 4 min were recorded. The cylinder was cleaned between rats. Rats tried to escape after dropping into the water. Irrespective of how much they struggled, they were trapped in the cylinder and eventually exhibited despair. The time of immobility or floating without movement represents the degree of susceptibility to depression.

#### 2.5.5 Conditional place preference (CPP) test

The CPP test was applied to reveal the presence of ongoing pain and reward from pain relief. CPP box consisted of two conditioning chambers distinguished by visual, tactile, and olfactory cues which were connected by a middle chamber. Time spent in conditioning chambers was recorded and analyzed by SuperMaze software (V 3.3.0.0, Shanghai Xinruan Information Technology Co., Ltd.). On the preconditioning day, rats had free access to all chambers for 15 min and the time spent in each chamber was recorded to exclude obvious chamber bias. On the conditioning day, rats were introduced to one chamber paired with the condition for 30 min. The positions of the chambers were switched 4 h later. On the test day, the time spent in the two chambers was recorded with rats having access to all chambers for 15 min. CPP scores were calculated as (C1 − C2) / (C1 + C2), where C1 is the time spent in the conditioned chamber and C2 is the time spent in the unconditioned chamber. Higher scores indicate a valid pain-relieving treatment and a strong preference for the specific chamber.

#### 2.5.6 Electroacupuncture

For acclimatization, rats were fixed in advance with a special cloth with acupoint area exposure. Ipsilateral acupoints of Zusanli (ST36, 5 mm below the fibula head, posterolateral of the knee joint) and Kunlun (BL60, the hollow area between the external malleolus and the tendon calcaneus) on the CFA-injected side were used in the LEA group. In the SEA + LEA group, the acupoints Baihui (GV20, medial parietal bone, 2 mm backward-oblique insertion) and Yintang (GV24+, midpoint of the eyebrows, 5 mm downward-flat insertion) were additionally used. Stainless steel EA needles (0.25 × 15 mm, Global, China) were connected to a Hans Acupoint Nerve Stimulator (HANS LH202H), the parameters of which were set as follows: 2/100 Hz, distant-dense wave, and stimulation intensities ranging from 0.5 to 1 mA (Liang et al., 2023). The treatment was given on seven consecutive days for 10 min each day.

#### 2.5.7 Immunofluorescence staining

Rats were deeply anesthetized with an intraperitoneal injection of 2% pentobarbital sodium (2 mL/kg) and transcardially perfused with 9% (w/v) saline followed by 4% (w/v) paraformaldehyde. The rat brains were subsequently extracted and post-fixed at 4°C for 3 days in paraformaldehyde. After dehydration in 20 and 30% sucrose, coronal sections of the VTA and NAc were sliced at a thickness of 15 μm on a freezing microtome (CM1900, Lecia, Germany). Cryosections were washed with TBST three times, blocked in 10% (wt/vol) normal goat/donkey serum at 37°C for 1 h, and then incubated overnight at 4°C with mouse monoclonal anti-tyrosine hydroxylase (anti-TH; 1:400, ab150659, Abcam), rat monoclonal anti-c-fos (1:800, ab214672, Abcam), and goat anti-GFP (1:2000, Abcam). Sections were then washed, incubated with Alexa Fluor 488- and Alexa Fluor 594-conjugated secondary antibodies for 1 h at room temperature, and finally incubated with 4,6-diamidino-2-phenylindole (ab104139, Abcam) for nuclear staining. Images were captured using a fluorescence microscope (BX53, Olympus).

#### 2.5.8 Western blotting

After being deeply anesthetized, rats were sacrificed by cervical dislocation. The VTA was quickly obtained and stored at −80°C. The fully milled fresh tissue was homogenized in the ice-cold buffer for 20 min, followed by a 15-min centrifugation at 12,000 rpm at 4°C. The protein concentration in the supernatant was determined by the BCA method. Boiled protein was separated by SDS-PAGE and transferred to PVDF membranes, which were then blocked by a mixture of TBST and skimmed milk for 1 h at room temperature. Membranes were incubated with anti-TH (1:4000, ab150659, Abcam) and anti-β-actin (1:1000, ab8227, Abcam) at 4°C overnight. After rinsing in TBST, membranes were incubated for 1 h at room temperature with goat horseradish peroxidase-conjugated IgG (1:5000, ab6721, Abcam). Target proteins were detected by enhanced chemiluminescence.

#### 2.5.9 Quantitative polymerase chain reaction (qPCR)

VTA tissues were homogenized and total RNA was extracted with TRIzol reagent. RNA concentrations were determined by a NanoDrop2000 spectrophotometer (Thermo). The extracted RNA was reverse-transcribed into complementary DNA (cDNA) using a cDNA synthesis kit (G3337, Beyotime Biotech Co., Ltd.). Gene expression levels were determined by a fluorescence qPCR instrument (CFX Connect, Bio-Rad) with the following reaction conditions: 95°C for 30 s, followed by cycles of 95°C for 15 s and 60°C for 30 s. To ensure accuracy, the measurement of each sample was performed in triplicate. The relative expression level of the TH gene was determined by the 2^−∆∆Ct^ method, while β-actin was used for normalization. The primers were obtained from Bingcure Biotech Co., Ltd. Primer sequences are shown in Table 1.

**Table 1:** Primer sequences.

Gene	Forward	Reverse
TH	5′-TCCTCCTTGTCTCGGGCTGTA-3′	5′-TTCCGACGCTGGCGATACA-3′
β-actin	5′-TGCTATGTTGCCCTAGACTTCG-3′	5′-GTTGGCATAGAGGTCTTTACGG-3′

### 2.6 Statistical analysis

Data are expressed as mean ± standard error and were analyzed using GraphPad Prism (version 9.5.0, GraphPad Software Inc., La Jolla, CA, United States) and SPSS (version 26). Data of PWTs and PWLs at different time points (baseline, 7 days, 21 days, and 28 days) were analyzed using repeated-measure analysis of variance (rmANOVA). For data of PWTs and PWLs at the same time point, Western blot and qPCR data, and data of behavioral tests including OFT, MBT, SPT, FST, and CPP scores, an independent t-test was performed when two groups were compared. For comparison of three groups, one-way ANOVA was performed followed by post-hoc multiple comparisons using the Tukey method. The time spent in the two chambers in the CPP test was analyzed using two-way ANOVA. Differences were considered statistically significant if *p* < 0.05.

## 3 Results

### 3.1 Fiber projection of VTA^DA^-NAc

**Figure 2:**
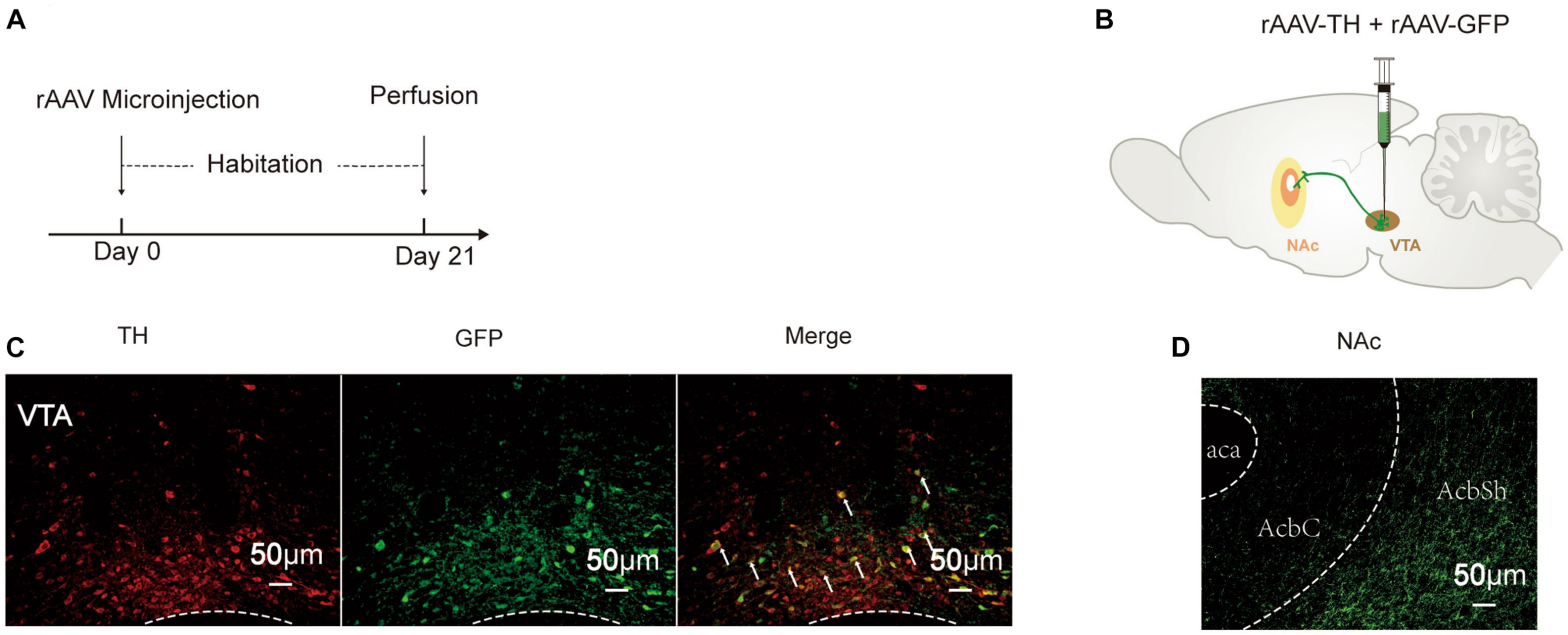
Fiber projection of VTA^DA^-NAc. **(A)** Experimental schedule. **(B)** Strategy of virus injection to label DA neurons in the VTA. **(C)** GFP and TH were co-localized in the VTA. **(D)** DA neurons projected to the NAc. rAAV-TH, rAAV-TH-CRE-flag-WPRE-pA; rAAV-GFP, rAAV-Efla-DIO-EGFP-WPRE-pA; DA, dopamine; VTA, ventral tegmental area; TH, tyrosine hydroxylase; NAc, nucleus accumbens; aca, anterior commissure, ant; AcbC, accumbens nu, core; AcbSh, accumbens nu, shell.

To determine the connectivity of the VTA and the NAc, 260 nL “rAAV-TH” and “rAAV-GFP” was injected successively into the VTA (Figures 2A,B). After 3 weeks of habituation, DA neurons were labeled by the GFP signal, and their fibers could be observed in the NAc (Figure 2D). TH, a marker of DA neurons, showed high co-expression with GFP (Figure 2C), indicating that the cells labeled by GFP were DA neurons. These results revealed that DA neurons project from the VTA to the NAc.

### 3.2 Emotion and reward disorders are comorbidities of hyperalgesia

**Figure 3:**
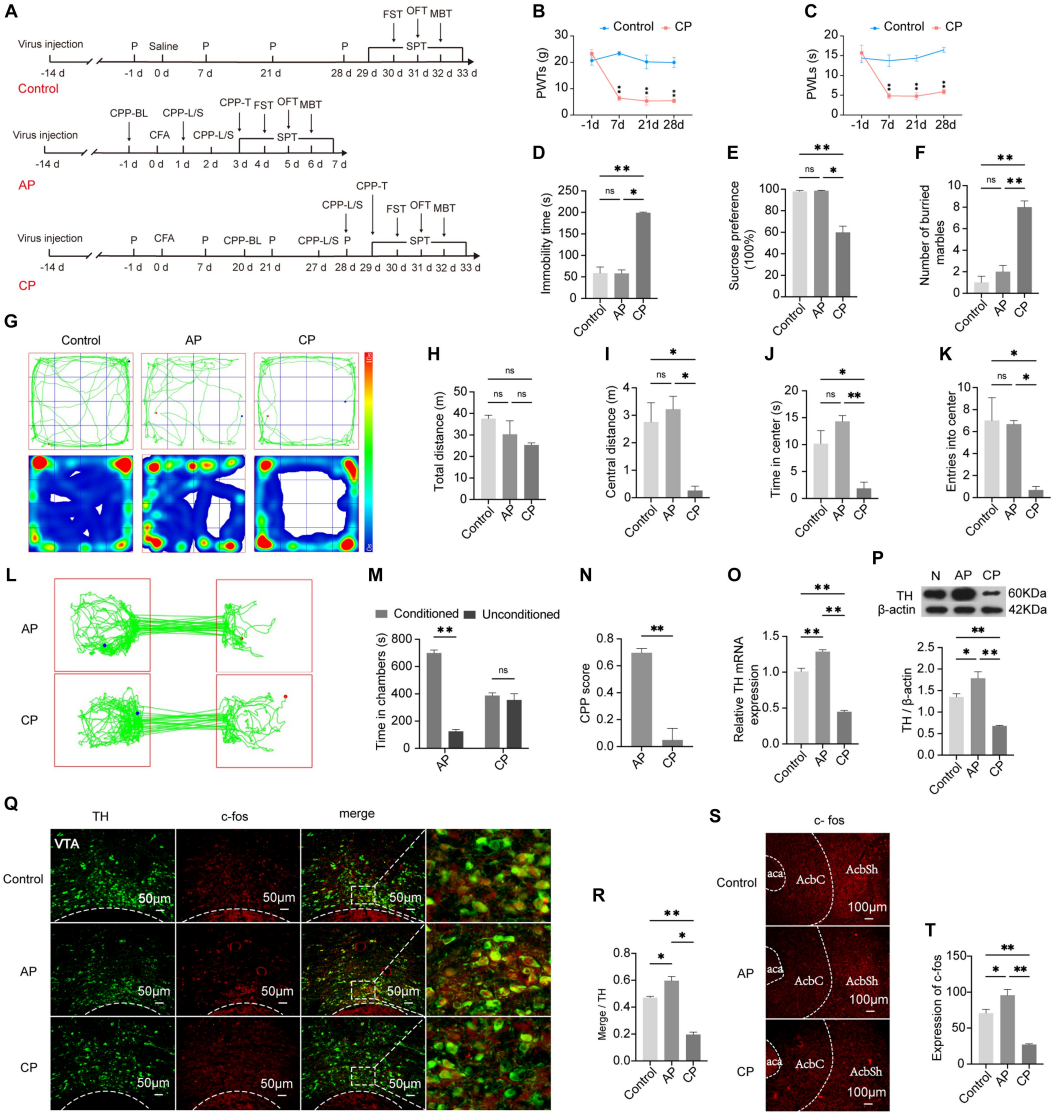
Negative emotions and suppressed reward behavior can be mediated by chronic pain. **(A)** The procedure of this part of the experiment. **(B)** PWTs in the control and CP groups at different time points. **(C)** PWLs in the control and CP groups at different time points. **(D)** Immobility time in the control, AP, and CP groups in the FST. **(E)** Preference for sucrose water in the SPT. **(F)** Number of buried marbles in the MBT. **(G)** Representative traveling tracks and heatmaps in the OFT. **(H)** The difference in total traveling distance in the OFT is not significant. **(I)** Traveling distance in the central area of the open field. **(J)** Time spent in the central area of the open field. **(K)** Times of entry into the center of the open field. **(L)** Representative exploratory tracks of rats in the AP and CP groups in the CPP test. **(M)** Time exploring the two chambers in the AP and CP groups in the CPP test. **(N)** Calculated CPP scores of the two groups. **(O)** The TH mRNA expression levels in the VTA among the three groups. **(P)** TH protein expression in the VTA in the three groups. **(Q)** Representative image showing the co-localization of TH and c-fos in the VTA. **(R)** Percentage of activated DA neurons in each group. **(S,T)** c-fos expression in the AcbSh. *p < 0.05, **p < 0.01. P, PWTs and PWLs; BL, baseline; CPP, conditional place preference; CPP – L/S, CPP was conditioned with lidocaine and saline; CPP – T, CPP – test; SPT, sucrose preference test; FST, forced swimming test; OFT, Open-field test; MBT, marble-burying test; AP, acute pain; CP, chronic pain; DA, dopamine; VTA, ventral tegmental area; TH, tyrosine hydroxylase; NAc, nucleus accumbens; aca, anterior commissure, ant; AcbC, accumbens nu, core; AcbSh, accumbens nu, shell.

CFA was injected two weeks after the virus injection in the VTA to avoid the influence on the behavior test. While the control group and the CP group were included to observe the relationship between chronic pain and negative emotions, the AP group was included to investigate the reward disorder in the chronification of pain (Figure 3A). Compared with rats that had received an intraplantar injection of saline, the 50% PWTs and PWLs of rats that had received a CFA injection were strongly reduced, and the pain threshold in the CP group was maintained at a significantly lower level, which is a characteristic of persistent pain (p < 0.01), confirming that CFA had successfully elicited inflammatory hyperalgesia (Figures 3B,C). Conversely, depression and anxiety-like behaviors of the CP group as assessed by the OFT, MBT, FST, and SPT were more obvious when compared with the AP group and the control group (Figures 3D–K). Besides, rats in the AP group spent more time in lidocaine-paired chambers and gained a higher CPP score compared with the CP group (*p* < 0.01) (Figures 3L–N), suggesting that the rats with chronic pain exhibited suppressed reward behavior. In comparison with the control group, co-expression of TH and the activity-dependent gene product c-fos in the VTA was increased in the AP group, while it was significantly decreased in the CP group (*p* < 0.05), which was consistent with the gene and protein expression of TH (Figures 3O–R). Moreover, c-fos expression in the medial AcbSh was higher in AP rats but lower in CP rats (*p* < 0.05), indicating increased activity of the VTA^DA^-NAc reward circuit in the AP group and decreased activity of the VTA^DA^-NAc reward circuit in the CP group. These results demonstrate that negative emotions and reward disorders appear in the late period after CFA injection but not in the early period, suggesting that emotional and reward dysfunction can be influenced by persistent pain, the mechanism of which may be related to the suppression of the VTA^DA^-NAc circuit.

### 3.3 Activation of the VTA^DA^-NAc circuit leads to attenuation of hyperalgesia and emotion and reward disorders

**Figure 4:**
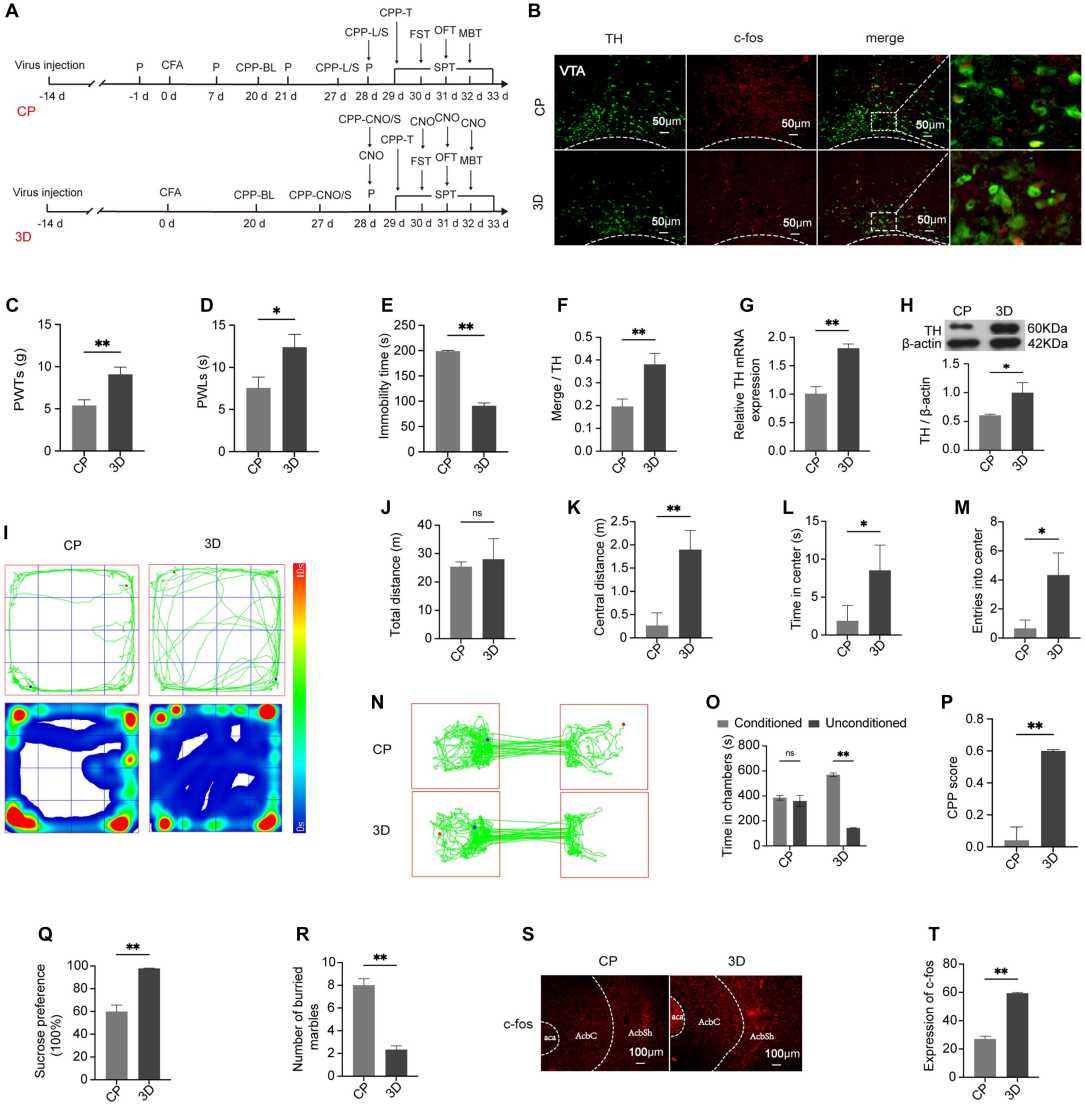
Hyperalgesia, negative emotions, and reward disorder can be alleviated through activating the VTA^DA^-NAc circuit. **(A)** Schematic showing the experimental procedure. **(B)** Representative images of labeled TH and c-fos in the VTA in the CP and 3D groups. **(C,D)** PWTs and PWLs. **(E)** Time of immobility in the FST. (F) Percentage of DA neurons expressing c-fos. **(G)** mRNA expression of TH in the two groups. **(H)** The expression level of TH. **(I)** Traveling tracks and heatmaps in the CP and 3D groups. **(J)** Total distance traveled in the OFT. **(K)** Distance covered in the center in the OFT. **(L)** Time spent in central areas in the OFT. **(M)** Times of entry into the central area in OFT. **(N)** Representative tracks of rats in the conditioning box. **(O)** Time spent in the two conditioning chambers in the CPP test. **(P)** CPP scores of the CP and 3D groups. **(Q)** Preference for sucrose in the SPT. **(R)** Quantification of marbles buried by rats within 15 min. **(S,T)** The expression of c-fos in AcbSh in the two groups. *p < 0.05; **p < 0.01. P, PWTs and PWLs; BL, baseline; CNO, clozapine-N-oxide; CPP, conditional place preference; CPP – T, CPP – test; SPT, sucrose preference test; FST, forced swimming test; OFT, Open-field test; MBT, marble-burying test; CPP – CNO/S, rats conditioned with CNO and saline in the CPP test; DA, dopamine; VTA, ventral tegmental area; TH, tyrosine hydroxylase; NAc, nucleus accumbens; aca, anterior commissure, ant; AcbC, accumbens nu, core; AcbSh, accumbens nu, shell.

To directly investigate the potential mechanism of the correlation between hyperalgesia and emotion and reward disorders, rats in the 3D group were stereotactically injected with “rAAV-TH” and “rAAV-3D-GFP” into the VTA, followed by intraplantar CFA injection 14 days later (Figure 4A). Since an increased quantity of DA neurons was co-labeled with c-fos, 3D rats showed significant activation of DA neurons in the VTA (*p* < 0.01) (Figures 4B,F). In correspondence, the mRNA and protein expression levels of TH were upregulated (Figures 4G, H). The activity of the medial AcbSh correspondingly reached a higher level in 3D rats (*p* < 0.01) (Figures 4S,T), suggesting increased activity of the VTA^DA^-NAc circuit by chemogenetic activation. The pain-like behavior characterized by 50% PWTs and PWLs was ameliorated in the 3D group compared with the CP group (*p* < 0.05) (Figures 4C,D). Exploration of central zones was increased in the 3D group, while the total time remained unchanged (Figures 4I–M). Similarly, the number of marbles buried within 15 min was obviously reduced, revealing the mitigation of anxiety-like behaviors (Figure 4R). In the 6-min FST, 3D rats were observed to spend significantly less time in a posture of immobility (Figure 4E). When provided with pure water and 1% sucrose solution *ad libitum*, 3D rats presented increased consumption of 1% sucrose solution (Figure 4Q). hM3D rats spent more time in the chamber pairing with CNO and the CPP scores were much higher than those among model rats (Figures 4N–P). These results revealed that hyperalgesia and emotion and reward disorders of CP rats could be reversed by activation of the VTA^DA^-NAc circuit.

### 3.4 Lea + SEA targeting the VTA^DA^-NAc pathway to enhance the alleviating effect of LEA on hyperalgesia and associated negative emotions

**Figure 5:**
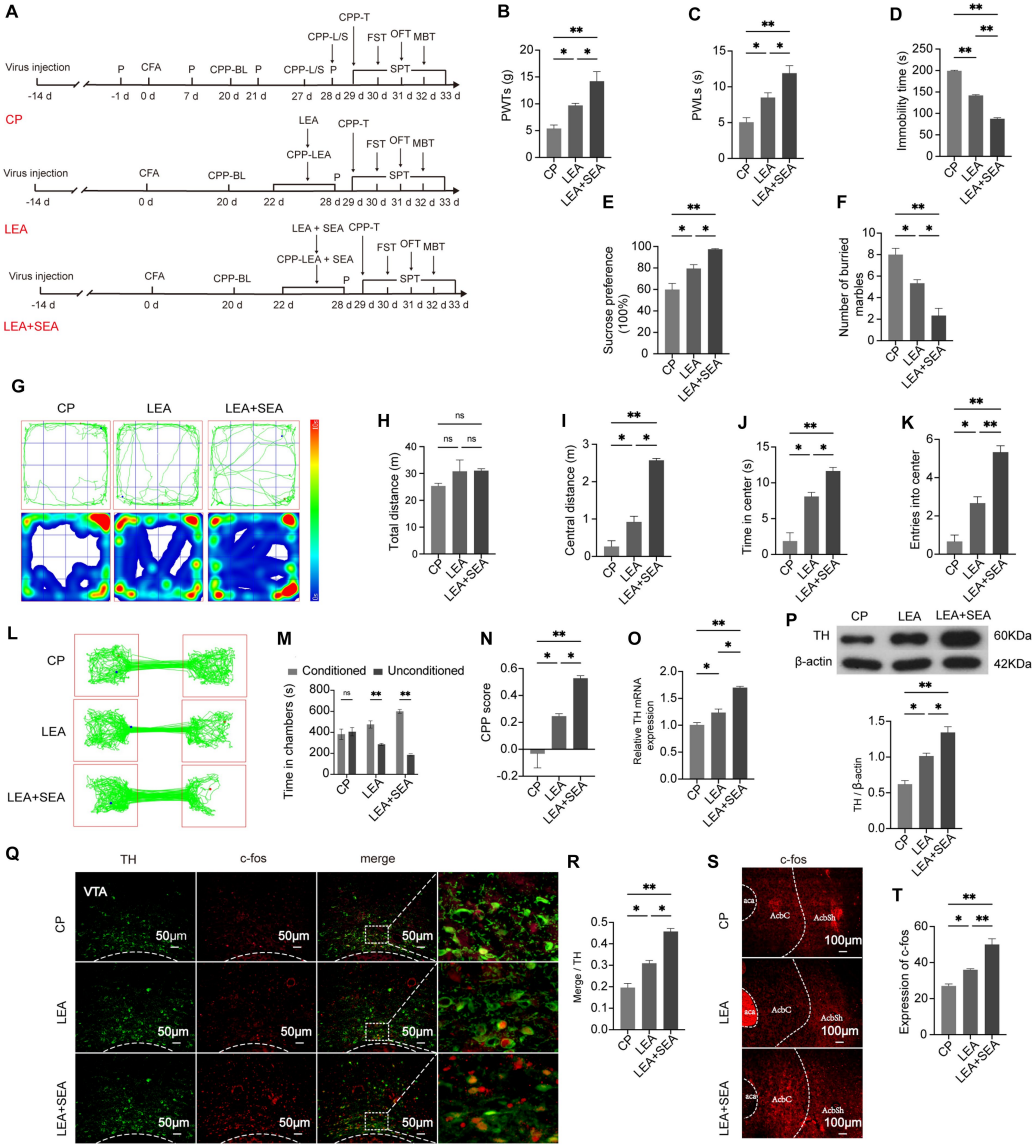
LEA + SEA is a preferred option in the treatment of hyperalgesia and associated comorbidities. **(A)** Protocol for this part of the experiment. **(B)** Mechanical pain threshold in response to Von Frey hairs. **(C)** PWLs of rats on the hot plate. **(D)** Time of immobility in the FST. **(E)** Preferences for sucrose water in the CP, LEA, and LEA + SEA groups. **(F)** Number of buried marbles in the MBT. **(G)** Representative rat track and heatmap in the OFT. **(H)** Total distance traveled in the OFT. **(I)** Distance traveled in the central area in the OFT. **(J)** Time traveled in the central area of the OFT. **(K)** Rat entries into the central area of the OFT. **(L)** Representative rat tracks in the CPP test. **(M)** Time spent in the two conditioning chambers. **(N)** CPP scores of each group. **(O)** Representative image for co-labeled TH and c-fos. **(P)** Percentage of DA neurons co-labeled with c-fos in each group. **(Q)** mRNA expression of TH. **(R)** Protein expression of TH. **(S,T)** c-fos expression in the AcbSh. *p < 0.05; **p < 0.01. P, PWTs and PWLs; BL, baseline; CPP, conditional place preference; CPP – T, CPP – test; SPT, sucrose preference test; FST, forced swimming test; OFT, Open-field test; MBT, marble-burying test; LEA, lower limb electroacupuncture; SEA, head electroacupuncture; CPP – LEA, rats are conditioned with LEA in the CPP test; CPP – LEA + SEA, conditioning chamber is paired with LEA + SEA; DA, dopamine; VTA, ventral tegmental area; TH, tyrosine hydroxylase NAc, nucleus accumbens; aca, anterior commissure, ant; AcbC, accumbens nu, core; AcbSh, accumbens nu, shell.

At day 21 post-CFA, LEA and LEA + SEA were given to CP rats (Figure 5A). After treatment for seven days, 50% PWTs and PWLs indicated pain-like behaviors were lower in the LEA group compared with CP rats (Figures 5B,C). Meanwhile, the analgesic effect of LEA was enhanced by LEA + SEA (*p* < 0.05) (Figures 5B,C). Based on the time spent in central zones in the OFT, marbles buried in the MBT, the time of immobility in the FST, and sucrose solution consumption in the SPT (Figures 5D–K), the anxiety and depression-like behaviors were improved in LEA rats, while the LEA + SEA rats showed an even more significant improvement (*p* < 0.01). Compared with CP rats, the time spent in chambers paired with EA treatment and the CPP score were obviously increased in LEA rats. Meanwhile, the LEA + SEA rats showed an even higher preference for EA-paired chambers, indicating that LEA + SEA is more beneficial than LEA alone (Figures 5L–N). Interestingly, the VTA^DA^-NAc pathway was significantly activated in LEA + SEA rats, which was found to be highly consistent with the CPP level (Figures 5O–T). These results revealed that LEA could alleviate hyperalgesia and associated negative emotions while LEA + SEA promotes the LEA effect targeting the VTA^DA^-NAc pathway.

### 3.5 Inhibition of the VTA^DA^-NAc pathway attenuates the effect of LEA + SEA on hyperalgesia and associated negative emotions

**Figure 6:**
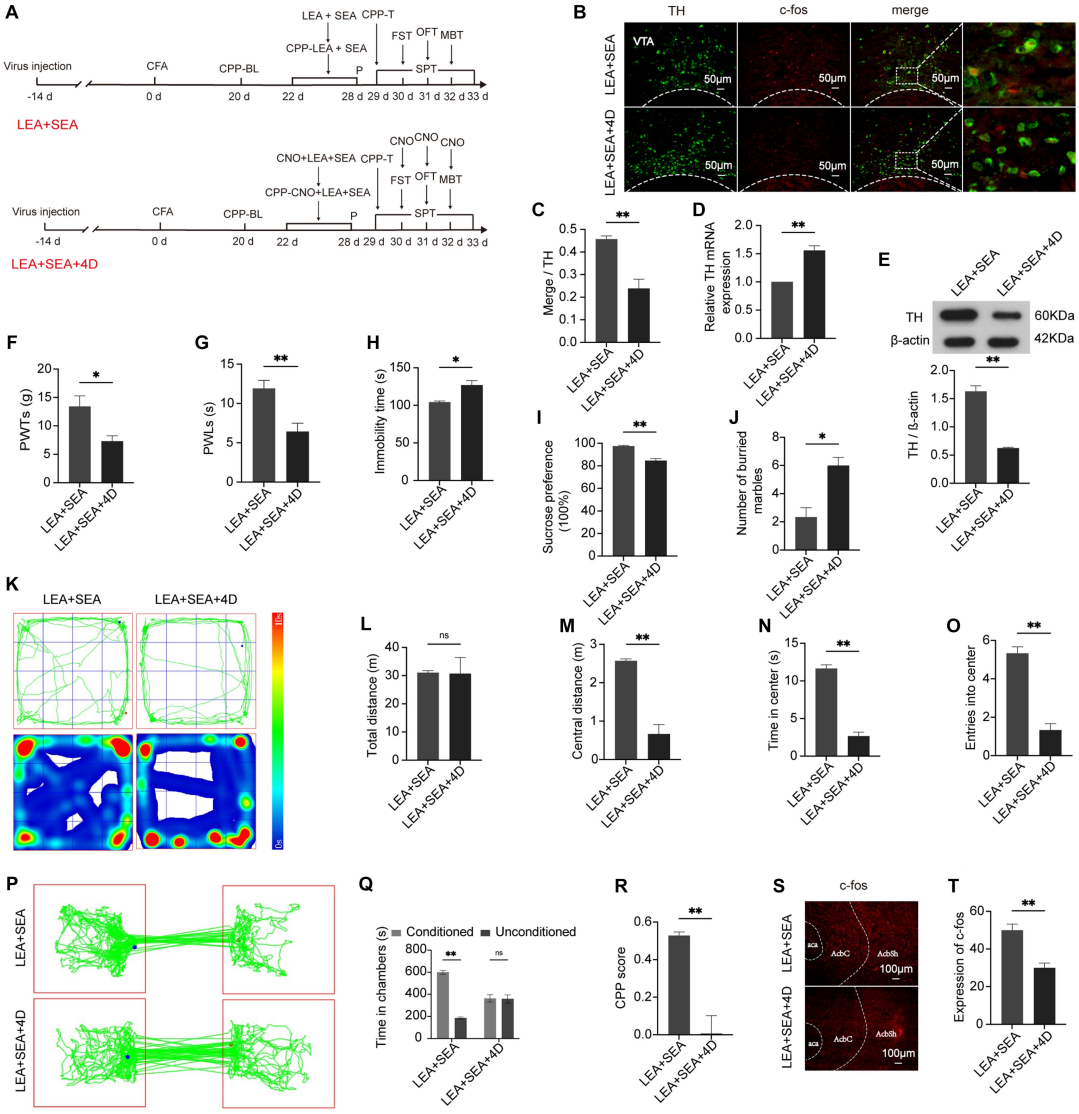
The effect of LEA + SEA can be attenuated by inhibiting the VTA^DA^-NAc circuit. **(A)** Schedule of this part of the experiment. **(B)** Representative image of TH co-labeling with c-fos in the VTA. **(C)** Percentage of DA neurons expressing c-fos. **(D)** mRNA expression of TH. **(E)** Relative protein expression of TH. **(F)** PWTs are influenced by inhibition of DA neurons. **(G)** PWLs in response to the hot plate. **(H)** Immobility of rats in the FST. **(I)** Preference for sucrose in the two groups. **(J)** Number of buried marbles in the MBT. **(K–O)** Behavior analyzed by the OFT. (P) Representative traveled trajectory in the CPP test. **(Q)** Time spent in two conditioning chambers. **(R)** CPP scores of the two groups. **(S,T)** The expression of c-fos in the AcbSh. *p < 0.05; **p < 0.01. P, PWTs and PWLs; BL, baseline; CNO, clozapine-N-oxide; CPP, conditional place preference; CPP – T, CPP – test; SPT, sucrose preference test; FST, forced swimming test; OFT, Open-field test; MBT, marble-burying test; LEA, lower limb electroacupuncture; SEA, head electroacupuncture; CPP – CNO + LEA + SEA, conditioning chamber is paired with CNO + LEA + SEA; DA, dopamine; VTA, ventral tegmental area; TH, tyrosine hydroxylase; NAc, nucleus accumbens; aca, anterior commissure, ant; AcbC, accumbens nu, core; AcbSh, accumbens nu, shell.

To address whether the VTA^DA^-NAc circuit can be specifically regulated by LEA + SEA, we delivered a chemogenetic fragment encoding DREADD hM4D + EGFP under the control of the TH promoter into rats to inactivate DA neurons in the VTA, which could not produce a marked effect without CNO. Two weeks after virus application, CFA was injected (Figure 6A). The VTA and NAc showed decreased c-fos expression in the LEA + SEA + hM4D group compared with the LEA + SEA group (*p* < 0.01) (Figures 6B,C). Correspondently, the mRNA and protein expression of TH displayed a significant downregulation in the LEA + SEA + hM4D group (Figures 6D,E). Moreover, protein expression of c-fos was obviously decreased in the LEA + SEA + hM4D group (*p* < 0.01) (Figures 6S,T), indicating a suppressed VTA^DA^-NAc pathway. Hyperalgesia, anxiety, and depression-like behaviors as well as reward disorder elicited by CFA were more obvious in the LEA + SEA + hM4D group (Figures 6F–R). Therefore, our results demonstrated that LEA + SEA alleviated CFA-elicited disorders, whereas the effect of LEA + SEA was attenuated by VTA^DA^-NAc inhibition.

## 4 Discussion

The process of pain sensitization occurs at three levels: dorsal horn circuits, the brainstem’s descending pain modulatory system, and the brain’s sensory signaling system. The last level is associated with cognitive, emotion, and reward-related processes (Tracey and Mantyh, 2007; Peirs et al., 2015; Yang and Chang, 2019). The shared brain regions such as the anterior cingulate cortex (ACC), the lateral habenular nucleus (LHb), and the ventromedial prefrontal cortex (vmPFC) are reported to enable chronic pain signals to transform into signals of negative emotions (Zhuo, 2016; Li et al., 2017; Liang et al., 2020). Previous research has also demonstrated that the activity of DA neurons via the VTA is related to both chronic pain and negative emotions (Taylor et al., 2015; Watanabe and Narita, 2018). Contrary to the traditional perspective that DA neurons in the VTA are exclusively in charge of reward perception, they also respond to the aversive aspects of events (Cohen et al., 2012), and dysfunction of the mesolimbic DA system is suggested to be a critical factor for the formation of chronic pain-associated depression and anxiety (Watanabe and Narita, 2018; Fox and Lobo, 2019). Functional magnetic resonance imaging (fMRI) data revealed that reduced VTA–striatal connectivity could be observed in patients with major depressive disorder (Kumar et al., 2018). This indicates that the suppressed mesolimbic DA reward system is related to chronic pain-induced negative emotions. As the brain circuits processing aversive aspects of pain overlap with reward circuits (Navratilova et al., 2016), the low DA state resulting from chronic pain has been supposed to be a key determinant of suppressed reward circuits (Serafini et al., 2020). It has become increasingly evident that persistent pain leads to decreased DA release in the ventral striatum (Taylor et al., 2015). Moreover, a previous study found that DA release can be enhanced by morphine, the effect of which can be suppressed in sciatic nerve ligation (Ozaki et al., 2002). Consistent with these findings, our results suggested that chronic pain leads to increased anxiety and depression-like behaviors, as well as decreased DA release from the VTA to the medial AcbSh. In addition, the CPP score, which is used to evaluate reward behavior, was decreased.

The mesolimbic DA system plays a crucial role in both analgesia and negative emotions. A study from 1983 first identified the efficacy of DA agents in analgesia (Dennis and Melzack, 1983). Noteworthily, social avoidance behavior in stress-susceptible mice could be reversed by antidepressant treatment with fluoxetine by regulating the release of DA in the mesolimbic system (Cao et al., 2010). Besides, evidence has suggested that negative emotions contribute to pain intensity (Skljarevski et al., 2009). One study suggested that relaxing music had analgesic effects by promoting the mood state (Lee, 2016). Consistent with this notion is the observation that pleasant odors have analgesic effects by regulating the activity of the NAc (Villemure et al., 2012). Thus, we hypothesized that the VTA^DA^-NAc circuit may interact with the effect of positive mood in analgesia. Our data showed that increasing the release of DA to the medial AcbSh by activating DA neurons in the VTA attenuates hyperalgesia and associated mood/reward disorders, supporting the above hypothesis.

Previous studies have revealed the potential of EA in the treatment of mood disturbances (Amorim et al., 2018; Yang et al., 2022). It is a suitable treatment for anxiety in Parkinson’s sufferers and depression in post-stroke ischemia patients (Li M. et al., 2017; Li Y. et al., 2017; Fan et al., 2022). In animal studies, EA was also indicated to help alleviate negative emotions caused by nicotine withdrawal (Chae et al., 2008). Even though the effects of EA on pain and its comorbidities have been demonstrated, they are mainly studied individually (Lin et al., 2020). Recent data showed that allodynia and emotional disorders could be effectively improved by EA but without considering the mood effect on analgesia (Wu et al., 2015). However, whether the addition of additional acupoints to regulate the mood state could promote the effectiveness of EA in alleviating hyperalgesia remains unclear. In previous studies, EA at acupoints GV20 and GV24+ was used to relieve negative emotions (Li et al., 2021; Yao et al., 2021). A rodent study found that the application of GV20 and GV24+ could effectively reduce the anxiety-like behavior induced by cocaine exposure (Nie et al., 2020). In the present study, LEA was applied to CP rats; our findings suggest that it could improve allodynia and its comorbidities. Notably, our research also provides novel evidence that allodynia can be further alleviated by LEA + SEA which was characterized by additional acupoints GV20 and GV24+ to LEA.

Studies have proved that EA can relieve pain and associated disorders by regulating the activity of various brain regions such as the prefrontal cortex, amygdala, insula, dorsal raphe nucleus, and cingulate cortex (Li et al., 2020; Jang et al., 2021). It was reported by clinical researchers that the symptoms of drug withdrawal including craving and negative emotions could be reduced by EA (Courbasson et al., 2007; Chen et al., 2018). While accumulating studies provide evidence for the role of the DA-mediated VTA-NAc circuit in drug addiction or drug withdrawal (Solinas et al., 2019; Poisson et al., 2021), EA has been proposed to reduce the behavior of “drug seeking” by normalizing the activity of the DA system (Lee et al., 2021). This indicates that EA may have a regulatory effect on DA release. Consistently, our results show that CP rats exhibited high activity of the VTA^DA^-NAc circuit after the application of LEA. Meanwhile, LEA + SEA was suggested to promote the effect of LEA on VTA^DA^-NAc pathway activation. Chemogenetic inhibition of the VTA^DA^-NAc circuit can suppress the effects of LEA + SEA, suggesting the activity of the VTA^DA^-NAc pathway is responsible for the effect of LEA + SEA on hyperalgesia and associated emotions. Our study demonstrated that the activity of the VTA^DA^-NAc circuit could be enhanced by acute pain but suppressed by chronic pain. Therefore, LEA may upregulate the activity of the VTA^DA^-NAc circuit by hindering the progression of pain. Nevertheless, the activation of the VTA^DA^-NAc circuit by LEA + SEA may partly be related to the relief of both chronic pain and negative emotions.

While the CPP paradigm is a proper method to evaluate the reward level which is indicative of the VTA^DA^-NAc circuit (Chen et al., 2018), the inducer varied in different groups in this study, which may lead to a certain deviation. Furthermore, it would be significant for future studies to record the activation level of the VTA^DA^-NAc circuit by electrophysiology.

**Figure 7:**
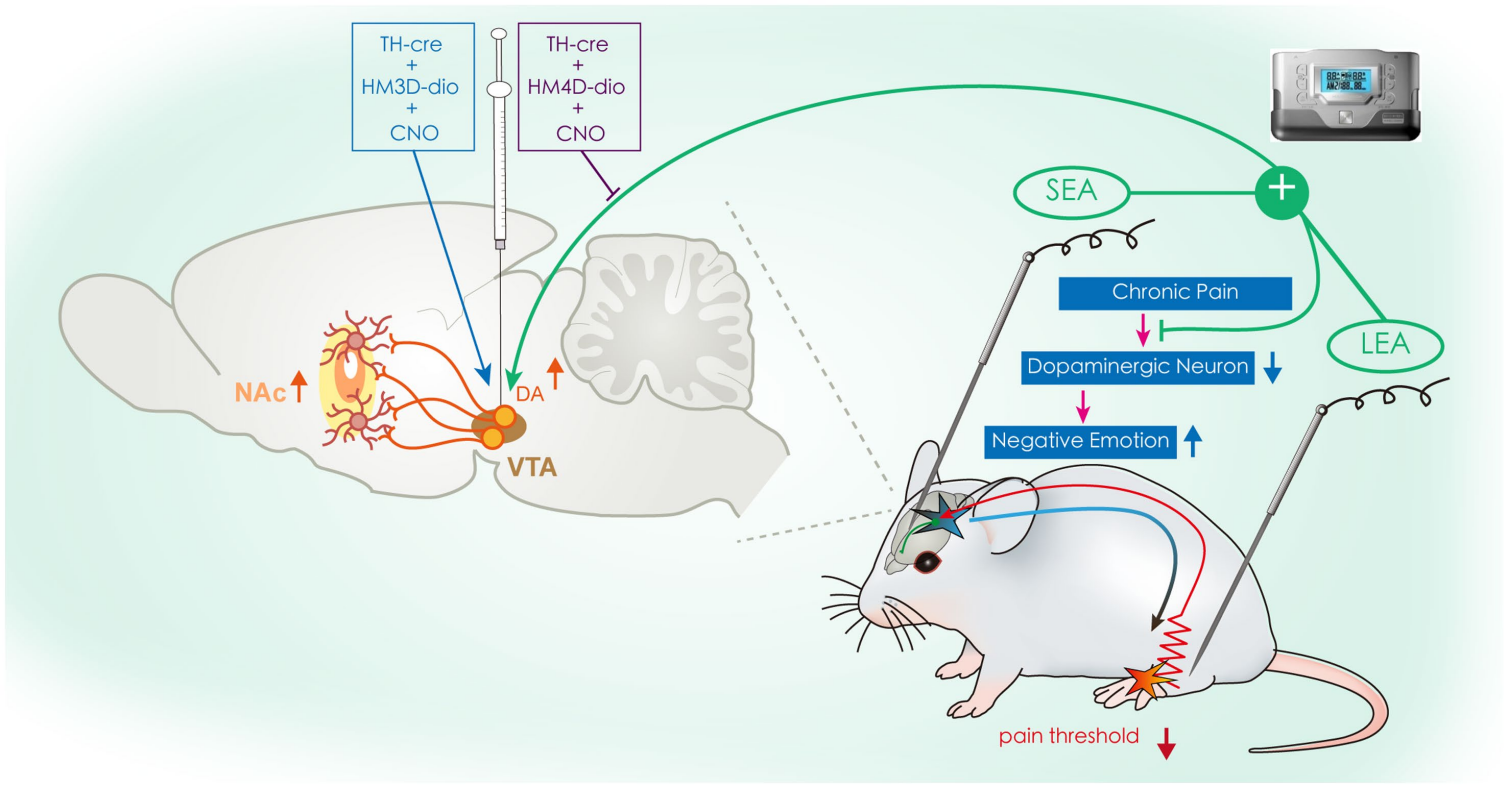
Schematic drawing illustrating the potential mechanism of SEA + LEA in treating chronic pain and associated negative emotions. VTA, ventral tegmental area; NAc, nucleus accumbens; DA, dopamine; SEA, head electroacupuncture; LEA, lower limb electroacupuncture; CNO, clozapine-N-oxide.

In conclusion, our results provide novel insight into the mechanism of EA in the treatment of chronic pain. Specifically, our experiments demonstrated that LEA + SEA is a preferred treatment in comparison with LEA alone for hyperalgesia and associated negative emotions by regulating the VTA^DA^-NAc pathway (Figure 7).

## Data availability statement

The data presented in the study are deposited in the Jianguoyun repository, the access link: https://www.jianguoyun.com/p/Dc-1umEQnYyeCRjhyKgFIAA.

## Ethics statement

The animal study was approved by Experimental Animal Welfare Ethics Committee, Zhongnan Hospital of Wuhan University. The study was conducted in accordance with the local legislation and institutional requirements.

## Author contributions

YY: Writing – review & editing, Funding acquisition. XW: Writing – original draft. JT: Supervision, Writing – review & editing. YZ: Writing – review & editing. JS: Writing – review & editing. QS: Writing – review & editing, Funding acquisition, Supervision.
